# Metabolic Enzyme Triosephosphate Isomerase 1 and Nicotinamide Phosphoribosyltransferase, Two Independent Inflammatory Indicators in Rheumatoid Arthritis: Evidences From Collagen-Induced Arthritis and Clinical Samples

**DOI:** 10.3389/fimmu.2021.795626

**Published:** 2022-01-17

**Authors:** Ming Lei, Meng-Qing Tao, Yi-Jin Wu, Liang Xu, Zhe Yang, Yan Li, Opeyemi Joshua Olatunji, Xiao-Wan Wang, Jian Zuo

**Affiliations:** ^1^ Xin’an Medicine Research Center, The First Affiliated Hospital of Wannan Medical College (Yijishan Hospital), Wuhu, China; ^2^ Research Center of Integration of Traditional Chinese and Western Medicine, Wannan Medical College, Wuhu, China; ^3^ Department of Rheumatology, The First Affiliated Hospital of Wannan Medical College (Yijishan Hospital), Wuhu, China; ^4^ Department of Traditional Chinese Medicine, The First Affiliated Hospital of Wannan Medical College (Yijishan Hospital), Wuhu, China; ^5^ Faculty of Traditional Thai Medicine, Prince of Songkla University, Hat Yai, Thailand; ^6^ Key Laboratory of Non-coding RNA Transformation Research of Anhui Higher Education Institution, Wannan Medical College, Wuhu, China

**Keywords:** triosephosphate isomerase 1 (TPI1), rheumatoid arthritis (RA), collagen-induced arthritis (CIA), metabolism reprogramming, glycolysis, monocytes, nicotinamide phosphoribosyltransferase (NAMPT)

## Abstract

Metabolic intervention is a novel anti-rheumatic approach. The glycolytic regulator NAMPT has been identified as a therapeutic target of rheumatoid arthritis (RA), while other metabolic regulators coordinating NAMPT to perpetuate inflammation are yet to be investigated. We continuously monitored and validated expression changes of *Nampt* and inflammatory indicators in peripheral while blood cells from rats with collagen-induced arthritis (CIA). Gene transcriptional profiles of *Nampt* ^+^ and *Nampt* ^++^ samples from identical CIA rats were compared by RNA-sequencing. Observed gene expression changes were validated in another batch of CIA rats, and typical metabolic regulators with persistent changes during inflammatory courses were further investigated in human subjects. According to expression differences of identified genes, RA patients were assigned into different subsets. Clinical manifestation and cytokine profiles among them were compared afterwards. *Nampt* overexpression typically occurred in CIA rats during early stages, when *iNos* and *Il-1β* started to be up-regulated. Among differentially expressed genes between *Nampt* ^+^ and *Nampt* ^++^ CIA rat samples, changes of *Tpi1*, the only glycolytic enzyme identified were sustained in the aftermath of acute inflammation. Similar to *NAMPT*, *TPI1* expression in RA patients was higher than general population, which was synchronized with increase in RFn as well as inflammatory monocytes-related cytokines like Eotaxin. Meanwhile, RANTES levels were relatively low when *NAMPT* and *TPI1* were overexpressed. Reciprocal interactions between TPI1 and HIF-1α were observed. HIF-1α promoted *TPI1* expression, while TPI1 co-localized with HIF-1α in nucleus of inflammatory monocytes. In short, although NAMPT and TPI1 dominate different stages of CIA, they similarly provoke monocyte-mediated inflammation.

## Introduction

The increase in prevalence of rheumatoid arthritis (RA) has continued to impose heavy burdens on the society and public health globally. RA affects people from all age groups, although its incidence of occurrence in older people is higher. The etiology of RA is yet to be fully elucidated. Several factors including infection, smoking, unhealthy diet and lifestyle, as well as genetic defects can contribute to its development (1). There are many RA diagnostic indicators available nowadays, such as rheumatoid factor (RF), C-reactive protein (CRP) and anti-cyclocitrulline antibodies (ACPA), but a substantial portion of patients are falsely diagnosed as negative according to these criteria (2). Meanwhile, several RA patients cannot benefit from standard anti-rheumatic regimens (3). Even initially effective therapies do not always maintain sustainable remission of RA. Above facts vividly demonstrate the difficulties in accurate diagnosis and successful treatment of this disease. Meanwhile, these clues indicate that RA is a heterogeneous disease. Under this background, defining RA subtypes will not only simply benefit the better understanding of their pathologies, but is also important for developing novel therapeutic strategies. It has been confirmed that CD4^+^ T cells play a vital role in RA (4). However, as a systematic autoimmune disease, RA etiology cannot be exclusively attributed to pathogenic T cells. Increasing RA-related immune cells are emerging, such as B lymphocytes, monocytes, macrophages, dendritic cells, natural killer cells and so on (5). As a result, differentiating RA subtypes by thoroughly characterizing their immune profiles is very difficult, considering our limited knowledge about immunity. To resolve this dilemma, more feasible and easier approaches should be introduced.

Many extra-articular manifestations of RA have been noticed. Cardiovascular diseases (CVDs) are believed to be the leading cause for RA-related pre-mature death, reflecting the disordered metabolism under this pathological condition (6). Consistent to this, dyslipidemia has been identified as one of the most common RA complications (7). Like osteoarthritis, overweight is a risk factor for RA. However, levels of most circulating lipids in RA patients like total cholesterol (T-CHO) and low density lipoprotein cholesterol (LDL-C) are typically lower than general population (8). Dramatic loss of body weight and fat reservoir usually indicates poor prognosis, and rheumatoid cachexia rarely occurs in obese subjects (9). Similarly, there is no consensus about blood glucose changes in RA patients until now, and whether diabetes is a RA complication is yet to be confirmed (10). These conflicting data suggest that RA patients should be categorized into different sub-cohorts, which have distinctly different metabolic profiles. It also hints that we should investigate RA-related metabolic changes horizontally and vertically. These seemingly contradictory observations would provide important clues for the better clarification of RA heterogeneity.

In Traditional Chinese Medicine (TCM) system, RA is divided into two subtypes (11). Patients with early/active RA tend to develop hot symptoms, which are characterized by obvious inflammation, and believed to be caused by pathogenic heat and dampness, two pathogens presumably related to accelerated energy metabolism (12). Cold symptoms are more common at later/inactive stages, when degradation and disability of joints become the dominate manifestations. This theory hints that energy metabolism varies a lot at different RA stages, and it is theoretically feasible to differentiate RA subtypes by monitoring metabolic statuses from the time dimension. Many metabolic regulators have been confirmed to be implicated in RA (13). Unfortunately, there are no studies on their dynamic changes.

Among these metabolic regulators, NAMPT is especially eye catching. Its product NAD^+^ is universally utilized as a co-enzyme in metabolism, and NAMPT itself has notable immunoregulatory functions (14). In brief, NAMPT is believed to promote M1 polarization of monocytes/macrophages by facilitating glycolysis (15). Its overexpression is usually observed under acute inflammation, and it is closely related to the intensified inflammation in rheumatic subjects (16, 17). We found that activating NAMPT exacerbated adjuvant-induced arthritis (AIA), and its down-regulation achieved the opposite outcome (17, 18). Considering the close relationship between NAMPT and inflammation, we assumed that this metabolic enzyme can be used to differentiate RA subtypes. In the current study, we continuously monitored *Nampt* expression in collagen-induced arthritis (CIA) rats. By comparing gene transcriptional profiles at different *Nampt* levels, we attempted to confirm that its overexpression is a hallmark of early/active CIA, and identify other possible metabolic regulators coordinating NAMPT during inflammation. Because RA is a systematic disease, circulating immune cells could have even more profound impacts than those resided in joints in perpetuating clinical manifestations. Besides, they are more accessible. Hence, the analyses were mainly based on white blood cells (WBCs) from peripheral blood and the corresponding plasma.

## Materials And Methods

### Chemicals and Reagents

Incomplete Freund’s adjuvant (IFA) and lyophilized immunization grade bovine type II collagen (CII) were obtained from Chondex (Redmond, WA, USA). Human CTACK, LIF, Eotaxin, RANTES, IL-17α, HIF-1α and IL-2R ELISA kits as well as rat IgG ELISA kit were supplied by Multi-Science (Hangzhou, Zhejiang, China). Colorimetric quantification kits used in ATP, blood glucose and triglyceride tests were purchased from Solarbio (Beijing, China). TRIzol Universal RNA extraction reagent and Universal qPCR Master Mix were procured from Keygen Biotech (Nanjing, Jiangsu, China) and New England Biolabs (Ipswich, MA, USA), respectively. Anti-human primary NAMPT, HIF-1α, TPI1, β-ACTIN antibodies, anti-rat primary Nampt, Tpi1, β-Actin antibodies, HRP-linked secondary antibodies and fluorescein-tagged secondary antibodies were provided by either ABclonal Technology (Wuhan, Hubei, China) or Affinity Biosciences (Changzhou, Jiangsu, China). APC-Cd86, PE-Cd206 and FITC-Cd11b antibodies together with ReverAid First Strand cDNA Synthesis kit were supplied by Thermo Fisher Scientific (Rockford, IL, USA). Resveratrol (RSV) and phosphoenolpyruvate (PEP) were bought from Sigma-Aldrich (St. Louis, MO, USA).

### Monitoring of Periodic Inflammation-Related Genes Expression in CIA Rats

All *in vivo* experiments were conducted in accordance with the National Institutes of Health Guide for the Care and Use of Laboratory animals (NIH Publications No. 8023, revised 1978) and approved by the Ethics Committee of Wannan Medical College (LLSC-2020-138). Twelve male rats (7 weeks old) supplied by Tianqin Biotechnology (Changsha, Hunan, China) were randomly divided into two groups. The rats were accommodated in a strictly controlled environment (temperature: 24 ± 2°C; relative humidity: 50 ± 2%; dark/light circle: 12 h) and had free access to tap water and standard rodent chow. After adaptive feeding, half of rats were injected with IFA-CII emulsion at hind left paw to induce CIA (18).

As polyarthritis typically develops 12-14 days after IFA-CII injection under normal conditions, we periodically collected peripheral blood through fossa orbitalis vein during day 10-30 in this preliminary experiment. Portion of the anticoagulation blood was used for complete blood cell count (CBC) on a PE-6800 VIT blood cell counter (Pukang Biotech, Shenzhen, Guangdong, China). The remaining blood was centrifugated to obtain plasma, which was used to determine IgG, glucose and triglyceride levels using appropriate kits in accordance with the manufacturers’ protocols. The cell precipitation was re-suspended in red blood cell lysis buffer (Solarbio, Beijing, China), and incubated at room temperature for 5 min. After erythrolysis, WBCs were obtained. Total RNA within these cells were exacted by TRIzol reagent, and used as templates to synthesize cDNA. Relative expression of gene *Nampt*, *Sirt1*, *Ppar-γ*, *iNos*, *Il-1β*, *Mcp-1*, *Arg-1*, *Il-10*, *Ifn-γ*, and *Il-17α* were assessed by RT-qPCR on a 7500 Real-Time PCR Detection system (Applied Biosystems, Life Technologies) using *β-Actin* as the internal reference based on 2^-ΔΔCT^ calculation. All the gene specific primers used in this study were synthesized by Sangon Biotech (Shanghai, China), and their sequences are included in **Supplementary S1**.

### Validation of Periodic Gene Expression Changes in CIA Rats

To confirm the preliminary results and avoid occasional factors-caused interferences, we replicated the experiment above. It is known that CIA severity is greatly affected by accommodation environment and rat strain. In this replicate experiment, we used male Wistar rats (7 weeks old) provided by the same vendor, and housed them in a Specific Pathogen Free (SPF) laboratory. The grouping and sampling arrangements were similar to those described above with slight modifications. The rats in CIA control group received a boost IFA-CII injection on day 7, and blood sampling started the next day to cover pre-clinical stages of CIA. We selectively investigated the expression of gene *Nampt*, *Sirt1*, *Ppar-γ*, *iNos*, *Il-1β*, *Arg-1* and *Il-10* here using PCR method to highlight the role of monocytes in CIA.

### Screening of Genes Involved in Active CIA

The whole course of CIA is typically divided into 3 phases according to clinical manifestations, i.e. pre-clinical, active and inactive stages. Our recent study demonstrate that onset of CIA is accompanied with accelerated glycolysis (19). When inflammation reaches peak, the severity is spontaneously attenuated, and CIA enters the inactive phase. Because Nampt promotes inflammatory glycolysis, we thought *Nampt* up-regulation could be an indicator of active CIA. Consequently, blood samples collected from every CIA rat in the replicate experiment were assigned into two categories according to *Nampt* expression. When *Nampt* was overexpressed, the blood was labeled as *Nampt* ^++^ sample. Shortly, *Nampt* expression was decreased to basal line (taking healthy controls as the reference). The sample collected at this time point was allocated to *Nampt* ^+^ category. *Nampt* ^++^ and *Nampt* ^+^ samples were collected approximately on day 8 and 14, respectively. Due to individual variations, the sampling time varied a bit, usually in the range of 2 days.

Subsequently, gene expression profiles between *Nampt* ^++^ and *Nampt* ^+^ samples were compared by RNA-sequencing approach. RNA-sequencing service was provided by LC-Biotech (Hangzhou, Zhejiang, China). Samples from 4 CIA rats at both *Nampt* ^++^ and *Nampt* ^+^ statuses were used in this assay, and 3 samples from normal healthy controls were taken as references. Total RNA extracted by TRIzol reagent was quantified, and the quality was evaluated based on RNA integrity. Poly(A) RNA was enriched using Oligo (dT) Dynabeads (Thermo Fisher, CA, USA). The resulting RNA was subsequently fragmented and reverse-transcribed to cDNA, which was used as a template to synthesize U-labeled second-stranded DNA. After further modification and size-based screening, the products were amplified by PCR procedures. Finally, 2×150 bp paired-end sequencing was performed on an Illumina Novaseq™ 6000 sequencing system (San Diego, CA, USA). Interferences in raw files including adaptor reads, low quality data and undetermined bases were filtered out. The clean data obtained were mapped into rat genome with the aid of Cutadapt 1.9, and all the reads were assembled by the aid of StringTie 1.3.4d with default parameters. Using the processed data, the comprehensive transcriptome was constructed by Gffcompare 0.9.8. Levels of mRNA were assessed by the value of FPKM. The genes with mRNA fold change > 2 or < 0.5 and p value < 0.05 between the two groups were taken as differentially expressed, and subjected to GO (Gene Ontology) and KEGG (Kyoto Encyclopedia of Genes and Genomes) pathway enrichment analyses.

### Validation of Inflammation-Related Genes in CIA Rats

Differentially expressed genes were then manually checked. If there was obvious conflict concerning their expression within one set of samples, these data were abandoned. Statistical differences calculated based on low FPKM (< 10) were also deemed as unreliable. According to these criteria, *Nampt* overexpression-related differential genes were identified, which were believed to contribute to the altered immune situations at different stages, and participate in regulating inflammatory responses.

To test whether these genes can differentiate immune profiles in CIA rats and serve as indicators for CIA subtypes classification, we conducted the validation experiment. In this assay, CIA model group was comprised of 16 male Wistar rats, and another 8 healthy rats served as normal controls. All the animals were housed in a SPF lab, and CIA was induced using the same protocol described above. On day 20, blood was sampled. At this time point, acute inflammation had started to resolve. Due to individual variations, inflammatory signs in some rats were obviously reduced. Therefore, it should be taken as the turning point of active and inactive CA stages according to clinical manifestations. As *Nampt* was believed as a driving force of CIA-related inflammation, the rats were divided into two equal subgroups. All CIA samples were ranked according to *Nampt* expression, and allocated into *Nampt* ^++^ and *Nampt* ^+^ subsets. Levels of inflammatory indicators in *Nampt* ^++^ samples were supposed to be higher than *Nampt* ^+^ subsets.

### Analysis of Blood Samples From RA Patients

Following clinical samples-involved experiments were performed in accordance with the guidelines of Declaration of Helsinki, and the protocol was approved by the Institutional Ethics Research Committee of the First Affiliated Hospital of Wannan Medical College. Written informed consent was obtained from all participants. RA blood samples from 128 outpatients were collected at Yijishan Hospital from February 2021 to March 2021. Twenty-eight control samples from age-paired general population were collected from the physical examination center during the same period. Expression of gene *NAMPT* and *TPI1* in peripheral WBCs was assessed by PCR method. Monocytes were isolated from certain blood samples using the isolation kits provided by Solarbio (Beijing, China), which were lysed in RIPA buffer. Levels of protein NAMPT and TPI1 in the cells were assessed by immunobloting method.

Afterwards, 20 RA samples were chosen. Expression of *NAMPT* and *TPI1* in WBCs was assessed by PCR. The samples were then ranked based on *NAMPT* and *TPI1* expression. Taking the results as a reference, the samples were assigned into 4 groups (*TPI1* ^+^ *NAMPT* ^+^, *TPI1* ^+^ *NAMPT* ^++^, *TPI1^+^* ^+^ *NAMPT* ^+^ and *TPI1* ^++^ *NAMPT* ^++^). Cytokine composition differences among these groups were characterized by using a Bio-Plex Pro Human Cytokine Screening 48-plex Panel (Bio-Rad, Hercules, CA, USA). According to results from this preliminary assay, 81 RA blood samples with well documented clinical information were selected. By ranking gene expression, WBCs samples with neither high levels of *NAMPT* nor *TPI1* were taken as *NAMPT* ^+^ *TPI1* ^+^controls, and the remaining samples were assigned into either *NAMPT* ^++^ or *TPI1* ^++^ groups. Levels of certain cytokines in this larger cohort were investigated and compared by ELISA method. Relevance between differential genes/cytokines and RA severity was further tested by available clinical data.

### Characterizing the Role of TPI1 in Inflammation

Some RA monocytes were seeded into 6-well plates. After attachment, they were treated by gene-specific siRNA to silence *HIF-1α*. siRNAs were synthesized by Genepharma Co., Ltd. (Shanghai, China), and their sequences are as follows: negative control (NC), 5′-UUCUCCGAACGUGUCACGUTT-3′ (sense), 5′-ACGUGACACGUUCGGAGAATT-3′ (anti-sense); si-HIF-1α, 5′-CCAGCAGACUCAAAUACAATT-3′ (sense); 5′-UUGUAUUUGAGUCUGCUGGTT-3′ (anti-sense). Forty-eight hours later, the cells were harvested. Levels of intracellular TPI1 were investigated by immunobloting method. Some other RA monocytes were treated by TPI1 inhibitor PEP (0.5 mM) for 24 h *in vitro*. Afterwards, portion of the cells were collected for immunobloting assay, and the rest were used in intracellular ATP determination. Levels of HIF-1α in the medium were determined by ELISA method.

In immunofluorescence assay, monocytes isolated from either RA patients or healthy volunteers were seeded in a 24-wells plate. Certain cells were treated by RSV (20 μg/ml) for 24 h. After being treated by 4% paraformaldehyde and 1% bovine serum albumin, the cells were incubated with anti-human TPI1 and HIF-1α primary antibodies, followed by a further incubation with fluorescein-tagged secondary antibodies in dark. After extensively washing, DAPI staining was carried out. Fluorescent images were photographed by a Fluorescence microscope (Leica, Wetzlar, Germany).

For the purpose of co-immunoprecipitation (Co-IP) analysis, lysate of RA monocytes were co-incubated by either rabbit IgG or anti-human TPI1 antibody in the presence of protein A/G plus-agarose at 4°C overnight. The beads were washed five times with the lysis buffer, and finally boiled in SDS-PAGE loading buffer. Obtained samples were subject to immunobloting experiment to evaluate HIF-1α levels.

Peritoneal macrophages were collected from healthy male C57 mice according to procedures reported previously (20). The cells were cultured in 6-well plates. Two hours later, portion of the attached cells were stimulated by LPS (100 ng/ml) or in the combination with PEP (0.5 mM) for 12 h. Subsequently, the cells were collected after trypsin digestion, and stained by FITC-Cd11b and APC-Cd86 antibodies. After being fixed and permeabilized, the cells were further incubated with PE-Cd206 antibody for 30 min at room temperature. The stained cells were subsequently fed to a flow cytometer (FC500, Beckman) for quantitative analysis. Cd11b^+^Cd86^+^ and Cd11b^+^Cd206^+^ cells were identified as M1 and M2 macrophages, respectively.

### Statistical Analysis

In this study, data were typically expressed as mean ± standard deviation (SD). Based on one-way analysis of variance (ANOVA) coupled with Tukey *post hoc* test, statistical differences were considered as significant when p values < 0.05 or 0.01. The correlations among different parameters were indicated by Pearson correlation coefficient. All the statistical analyses were performed with GraphPad Prism 8.0 (Cary, NC, USA).

## Results

### NAMPT Overexpression Is an Event of Early CIA

**Figure 1:**
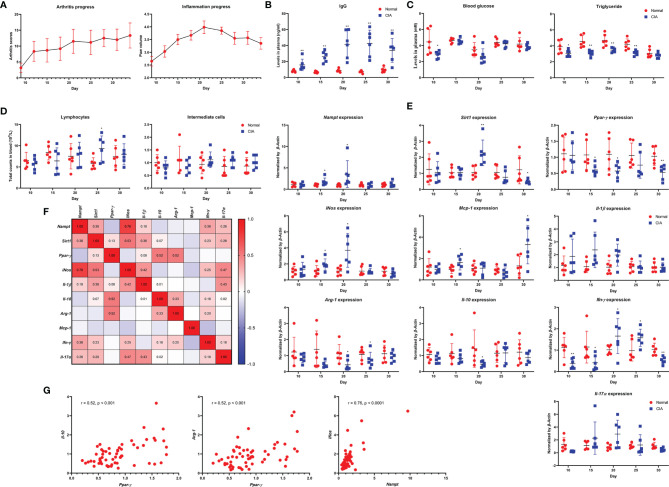
Dynamic changes of *Nampt* expression and inflammatory indicators throughout the course of CIA in male SD rats feed under normal environment. **(A)** periodic changes of arthritis scores and hind left paw volume; **(B)** IgG level changes in blood; **(C)** blood glucose and triglyceride level changes in blood; **(D)** lymphocyte and intermediate cell count changes in blood; **(E)** mRNA expression of *Nampt* and other inflammatory genes in peripheral WBCs; **(F)** expression correlations among different genes; **(G)** key correlations demonstrated in scatter plots diagrams. Statistical significance: ^*^p < 0.05 and ^**^p < 0.01 compared with normal healthy rats.

Arthritic manifestation gradually developed in male SD rats that received IFA-CII injection since day 12. In line with this, no obvious edema occurred in paws until then. Edema reached the peak about 8 days after the onset of polyarthritis, and was spontaneously attenuated thereafter (**Figure 1A**). Significant increase in IgG was noticed in CIA rats from the very beginning, and it was reinforced from day 20-25, lagging behind the progress of inflammation (**Figure 1B**). Blood glucose decrease was mainly observed before day 20. Meanwhile, levels of triglyceride in model animals were constantly lower than healthy controls until day 30 (**Figure 1C**). Lymphocyte and intermediate cell counts were generally decreased in CIA rats during day 10-15, while their population was recovered and even increased thereafter (**Figure 1D**). Morphological observation of paws and counts of other blood cells are shown in **Supplementary S2**. The progress pattern of CIA was further investigated by PCR analysis. Expression of *Nampt*, *iNos*, *Mcp-1* and *Il-1β* were obviously up-regulated in CIA rats during day 15-20, suggesting reinforced M1 polarization of monocytes in this period. M2 polarization was constantly deficient from the onset of polyarthritis as indicated by impaired expression of *Ppar-γ*, *Arg-1* and *Il-10*. As a downstream of *Nampt*, *Sirt1* was initially up-regulated. Probably due to the persistent deficiency of M2 polarization, *Sirt1* were down-regulated after day 25 (**Figure 1E**). Similar to IgG production, increase in expression of *Ifn-γ* and *Il-17α* mainly occurred during day 20-25. Collective PCR data show that *Nampt* strongly correlated to M1 polarization indicator *iNos*, while *Ppar-γ* exhibited good correlation with M2 polarization indicator *Arg-1* and *Il-10* (**Figure 1F**). These findings were subsequently illustrated in scatter plots diagrams (**Figure 1G**).

**Figure 2:**
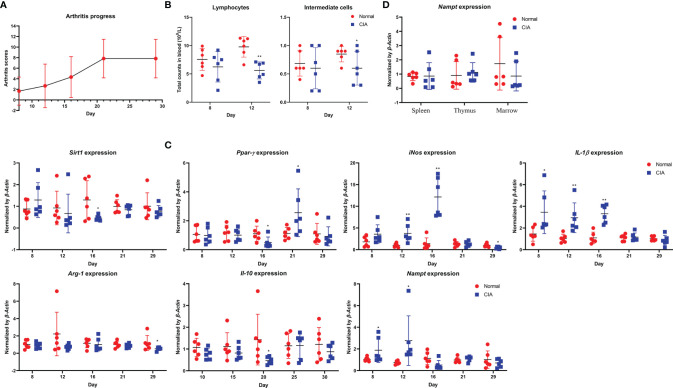
Validation of periodic changes in CIA using male Wistar rats housed in SPF laboratory. **(A)** periodic changes of arthritis scores; **(B)** decrease in lymphocyte and intermediate cell counts observed during early CIA; **(C)** periodic mRNA expression of *Nampt* and monocytes polarization-related genes in peripheral WBCs; **(D)** mRNA expression of *Nampt* in main immune organs of rats when sacrificed. Statistical significance: ^*^p < 0.05 and ^**^p < 0.01 compared with normal healthy rats.

In the replicate experiment, we therefore focused on monocytes-related changes. Despite the different rat strain and feeding environments, CIA progressed in a similar pattern (**Figure 2A**). Decreased counts of lymphocytes and intermediate cells were observed on day 8 and 12, indicating the early/active stages of CIA (**Figure 2B**) (21). Altered expression of *Sirt1*, *Ppar-γ*, *Arg-1*, *Il-10*, *Nampt*, *iNos* and *Il-1β* was observed in CIA rats once again (**Figure 2C**). The boost IFA-CII injection facilitated CIA development, as up-regulation of *Nampt* and *iNos* occurred earlier. We noticed that *Nampt* overexpression generally occurred prior to the onset of arthritis in these rats, and lasted for no more than 5 days typically. Since day 16, this phenomenon was reversed, and *Nampt* expression was even decreased (**Figure 2C**). In fact, no increase in *Nampt* expression in main immune organs of CIA rats was observed when the animals were sacrificed at day 30 (**Figure 2D**). Evidences from the two experiments collectively demonstrate that *Nampt* overexpression occur during early stages of CIA. Meanwhile, it questions the speculation that *Nampt* up-regulation is a hallmark of active CIA. Even in the preliminary experiment, its expression had been decreased below basal level before inflammation was resolved.

### Gene Transcriptional Profile Varies at Different CIA Stages

**Figure 3:**
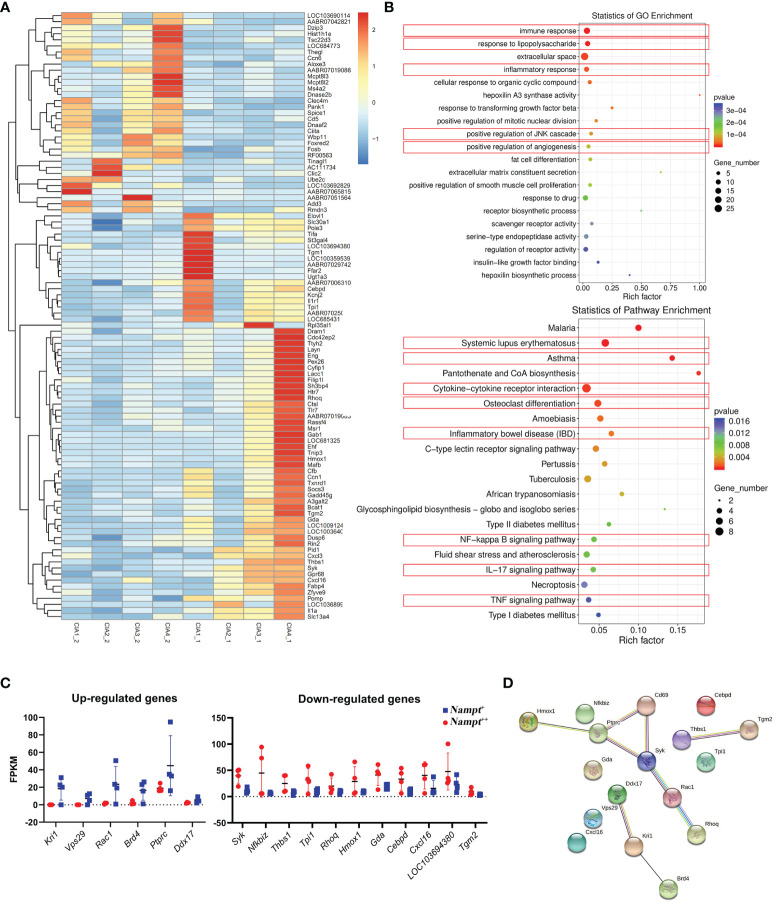
Screening of genes differentiating transcriptional profiles of CIA rats at different *Nampt* expression statues. **(A)** differentially expressed genes between *Nampt* ^++^ and *Nampt* ^+^ samples collected from identical rats revealed by RNA-sequencing; **(B)** results of pathway enrichment analyses using differentially expressed genes based on either GO or KEGG annotation, and those closely related to inflammation were highlighted in red; **(C)** manually selected genes with reliable changes; **(D)** interactions among selected differentially expressed genes/proteins.

To clarify key changes occur in early CIA characterized by *Nampt* overexpression, we performed RNA-sequencing using two batches of WBCs sampled from the same rats at different time points. Differentially expressed genes under *Nampt* ^++^ and *Nampt* ^+^ statuses were displayed in **Figure 3A**. GO enrichment demonstrates that the differed *Nampt* expression greatly affected immune status. Of note, lipopolysaccharide (LPS) response signaling (a key pathway-driven M1 polarization) was identified as one of the significantly affected pathways, confirming the relevance of *Nampt* overexpression to monocyte/macrophages-mediated inflammation. KEGG enrichment indicates that many immune disease pathways as well as related signal transductions were altered too (**Figure 3B**). The up-regulated and down-regulated genes (*Nampt* ^+^ vs *Nampt* ^++^ samples) were illustrated in **Figure 3C** separately. Interactions among corresponding proteins were visualized with the aid of an online tool STRING (www.string-db.org/). As shown in **Figure 3D**, most of the identified genes/proteins are involved in inflammatory network. Some remaining genes also play crucial roles in inflammation, such as *Nfkbiz*, *Cxcl16* and *Cebpd*, and the rest of them are typically categorized as metabolism regulators, including *Vps29*, *Gda* and *Tpi1*.

**Figure 4:**
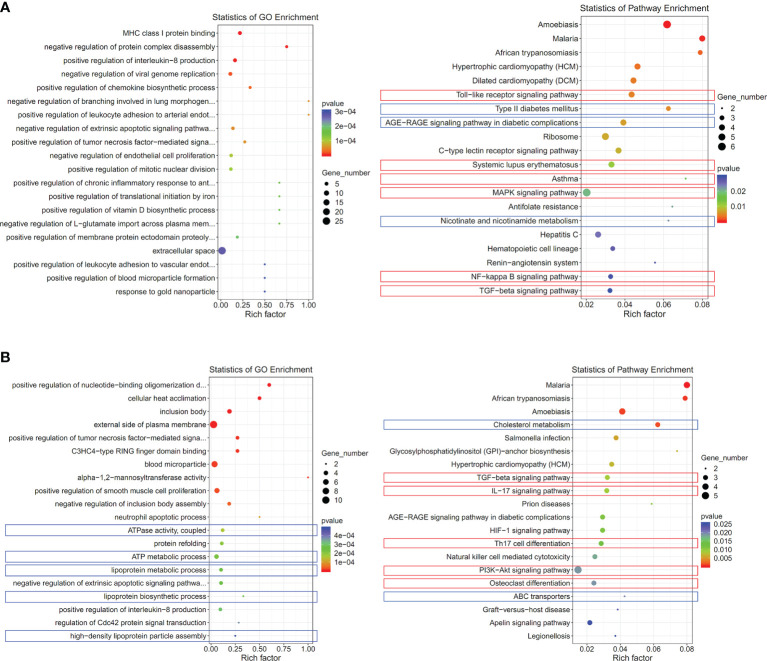
Significantly altered pathways in CIA rats at different *Nampt* expression statues. **(A)** results of pathway enrichment analyses using differentially expressed genes between CIA rats at *Nampt* ^++^ status and normal healthy controls; **(B)** results of pathway enrichment analyses using differentially expressed genes between CIA rats at *Nampt* ^+^ status and normal healthy controls. The altered pathways related to inflammation and energy metabolism were indicated in red and blue, respectively.

Subsequently, we screened out differentially expressed genes between healthy controls and CIA rats at both *Nampt* ^++^ and *Nampt* ^+^ statuses, which were listed in **Supplementary S3**. It was obvious that that high expression of *Nampt* was linked to acute inflammation, because TLRs and its downstream MAPKs and NF-κB were identified as the mostly affected targets in *Nampt* ^++^ CIA rats (**Figure 4A**). Interestingly, by the decrease of *Nampt* expression, the pathological situation became even more complicated. Energy metabolism (mainly lipid metabolism) profile was significantly changed. Many RA-related targets emerged, such as TGF-β, IL-17/IL-17 cell differentiation, PI3K-AKT and osteoclast differentiation pathways (**Figure 4B**). These results confirm that *Nampt* overexpression is mainly involved in activation of innate immune system in early CIA, while its down-regulation doesn’t necessarily mean the improved immune environment.

### *Tpi1* Perpetuates Inflammation in Active CIA

**Figure 5:**
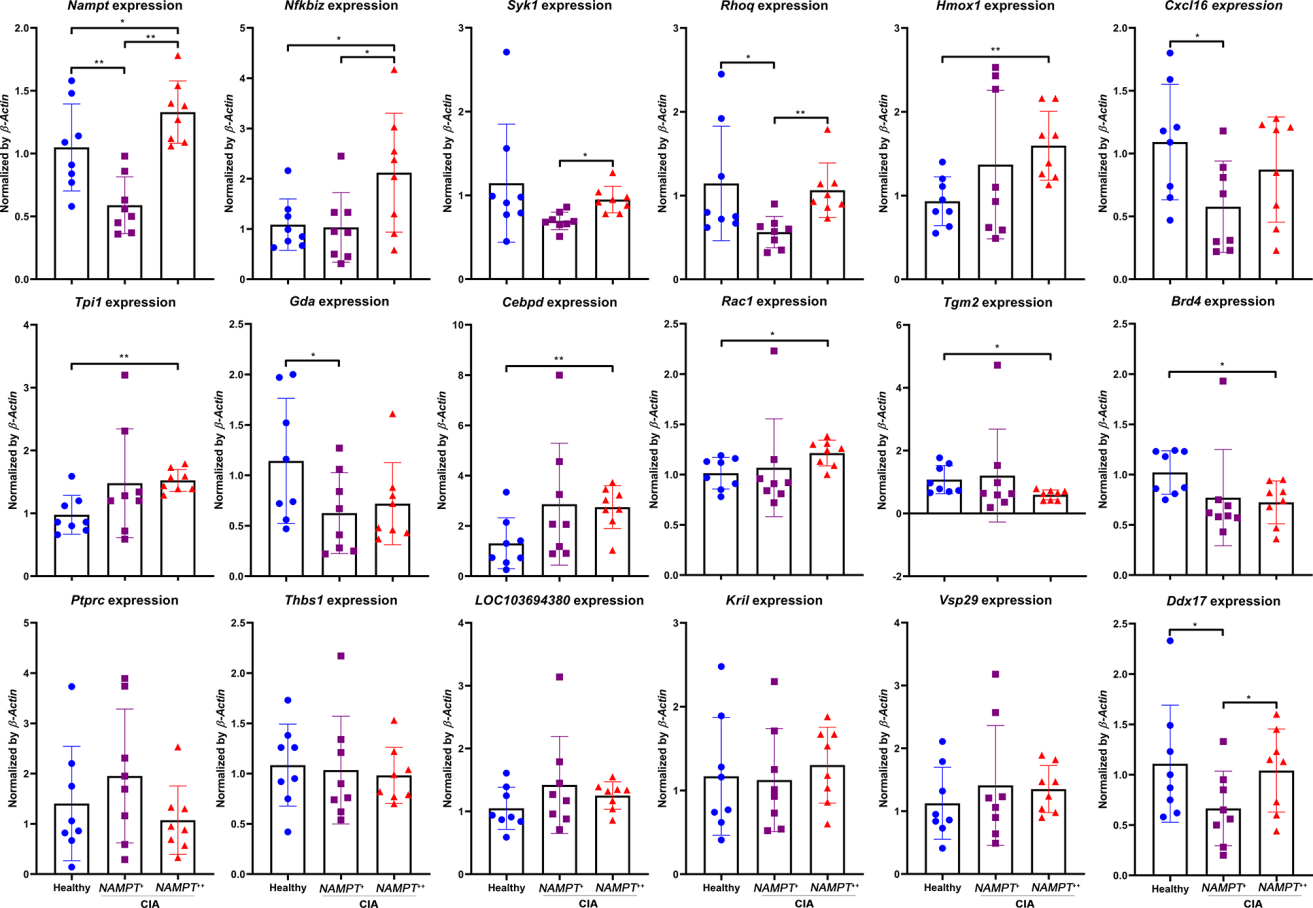
Expression of previously identified differentiation genes in WBCs isolated from either healthy or CIA rats on day 20. CIA rats were assigned into 2 subgroups according to relative *Nampt* expression. The statistical significance p values < 0.05* or 0.01** were calculated between groups matched by lines.

Although *Nampt* overexpression had been confirmed as a fact in early CIA, it was transient, which disappeared before the peak of inflammation. It is reasonable to assume that certain genes play even more important roles in perpetuating CIA-related inflammation. Metabolism reprogramming drives inflammation in CIA rats, which occurs even before arthritis onset (19). To sustain inflammatory aerobic glycolysis throughout the whole course of active CIA, persistent up-regulation of certain glycolytic regulators is very plausible and necessary. They may be similarly implicated in early CIA, and thereby included in the differential genes identified at *Nampt* ^++^ status. On day 20, *Nampt* overexpression in CIA rats had already disappeared, but we can still categorize the samples into two groups according to relative gene expression. Expression differences of many previously identified genes between healthy controls and CIA rats became not so obvious, including *Ptprc*, *Thbs1*, *LOC103694380*, *Kril*, and *Vsp29* (**Figure 5**). It suggests that these genes contribute less to the persistent inflammation in CIA rats, and they are also not sensitive to *Nampt* expression changes. Expression of *Nfkbiz*, *Syk1*, *Rhoq*, *Hmox1* and *Cxcl16* varied in a similar pattern between *Nampt* ^++^ and *Nampt* ^+^ samples to the phenomenon observed above. The constantly synchronized changes demonstrate that they are typical *Nampt-*related genes. As *Nampt* overexpression mainly occurs in early CIA, roles of these genes in the persistent inflammation would be similarly less important. The remaining genes including *Tpi1*, *Gda*, *Cebpd*, *Rac1*, *Tgm2*, *Brd4* and *Ddx17* were then categorized as *Nampt*-independent inflammation regulators in CIA. They closely link to chronic inflammation. Meanwhile, *Nampt* is not the decider of their status, because their expression did not always correlate to *Nampt* changes. We selectively focused on *Tpi1* afterward, as it is the only glycolytic regulator among them. It was significantly up-regulated in *Nampt* ^++^ CIA rats. But even in *Nampt* ^+^ CIA rats, its expression was still higher than healthy controls.

### *TPI1* Is an Inflammatory Indicator of RA Independent of *NAMPT*

**Figure 6:**
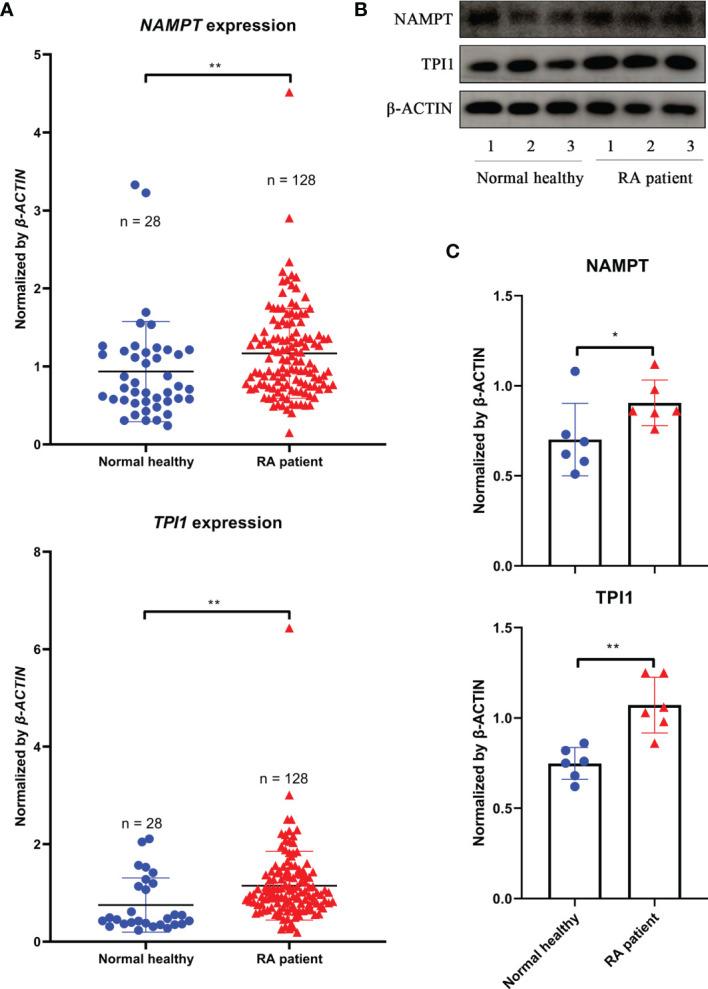
Differences of NAMPT and TPI1 levels between RA patients and general population. **(A)**, *NAMPT* and *TPI1* expression in WBCs samples; **(B)**, representative image of immunobloting assays evaluating NAMPT and TPI1 levels in WBCs isolated from RA patients and healthy volunteers; **(C)**, quantification results of immunobloting assay. The statistical significance p values < 0.05* or 0.01** were calculated between groups matched by lines.

Above evidences show that *Tpi1* is a *Nampt*-independent inflammatory metabolic regulator in CIA rats. To confirm this in RA, we performed following analyses using clinical samples. As expected, *NAMPT* expression in WBCs from RA patients was generally higher than general population, and so was *TPI1*. Compared with *NAMPT*, RA-related increase of *TPI1* was even more obvious (**Figure 6A**). Monocytes-based immunobloting analysis obtained similar results (**Figure 6B**). Despite individual variations, levels of both NAMPT and TPI1 in RA patients were higher than healthy volunteers (**Figure 6C**).

**Figure 7:**
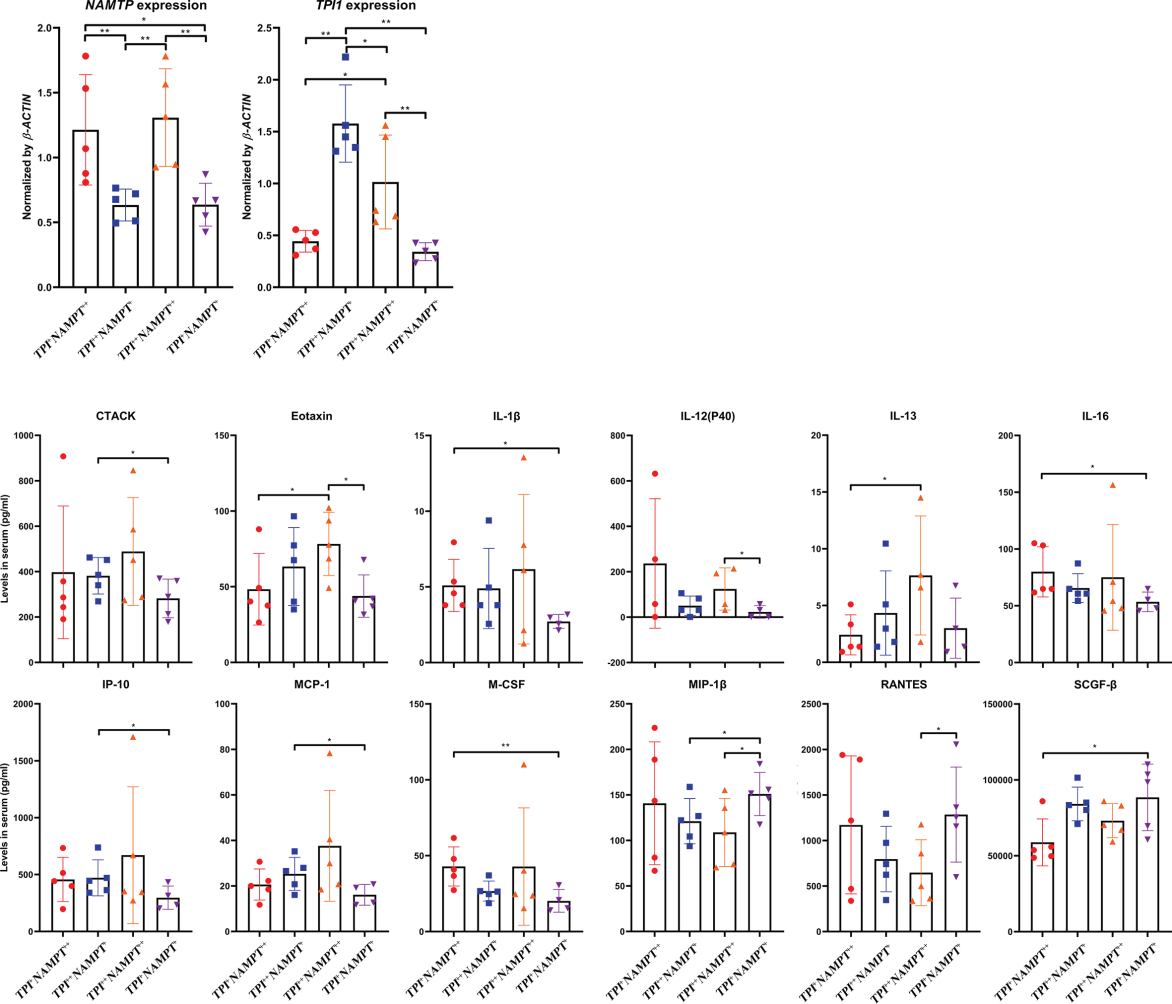
Impacts of *NAMPT* and *TPI1* expression on cytokine profile in RA patients. **(A)** grouping strategy and mRNA expression levels of *NAMPT* and *TPI1* in different RA subgroups; **(B)** levels of cytokines with significant concentration differences among subgroups. The statistical significance p values < 0.05* or 0.01** were calculated between groups matched by lines.

Afterward, we classified 20 RA samples according to *NAMPT* and *TPI1* expression, and compared their cytokine profiles. In total, there were 4 RA groups (*NAMPT* ^+^ *TPI1* ^+^, *NAMPT* ^++^ *TPI1* ^+^, *NAMPT* ^+^ *TPI1* ^++^ and *NAMPT* ^++^ *TPI1* ^++^). Incidence of typical NAMPT^++^Tpi1^++^ cases was low, and some NAMPT^++^ samples with relative high expression of *TPI1* were allocated into this category. As a result, *TPI1* expression in *NAMPT* ^++^ *TPI1* ^++^ subset was lower than *NAMPT* ^+^ *TPI1* ^++^ counterpart (**Figure 7A**). The 48 cytokines tested and their levels in samples were listed in **Supplementary S4**. The cytokines with significant concentration differences among groups were illustrated in **Figure 7B**. Compared with *NAMPT* ^+^ *TPI1* ^+^ controls, significant changes about levels of CTACK, Eotaxin, IL-1β, IL-13, IP-10, MCP-1, MIP-1β and RANTES were observed in *NAMPT* ^++^ *TPI1* ^++^ samples. It suggests that collective overexpression of *NAMPT* and *TPI1* could indicate the intensified inflammation. It is worthy of notice that all the increased cytokines/chemokines identified in *NAMPT* ^++^ *TPI1* ^++^ samples are involved in polarization/chemotaxis of M1 monocytes. On the opposite, RANTES a potent chemokine for inflammatory T cells enrichment was decreased (22). Although similar changes were observed in *NAMPT* ^++^ *TPI1* ^+^ and *NAMPT* ^+^ *TPI1* ^++^ subsets, their cytokine profiles were not exactly the same. Comparatively, *NAMPT* ^+^ *TPI1* ^++^ samples showed higher levels of Eotaxin, IL-13 and MCP-1 (**Figure 7B**). In short, *NAMPT* ^+^ *TPI1* ^+^ RA samples showed the lowest levels of M1 phenotype cytokines.

### *TPI1* and *NAMPT* Have Common Effects on RA Manifestation

**Figure 8:**
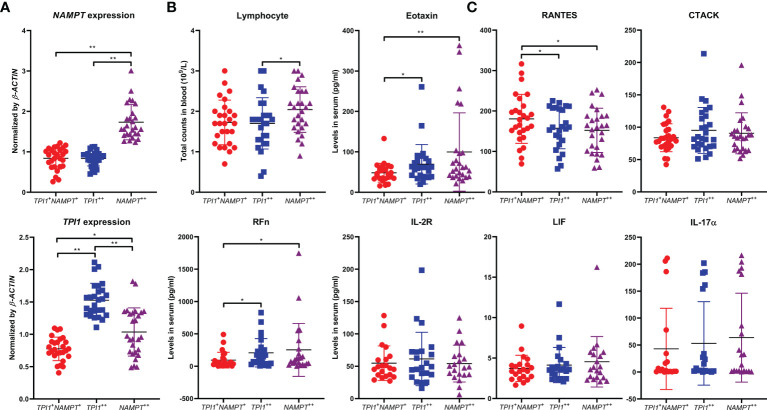
Similar impacts of *NAMPT* and *TPI1* overexpression on RA manifestation. **(A)** overview of *NAMPT* and *TPI1* expression in WBCs from different RA subgroups; **(B)** differences of lymphocyte count and RFn concentration among subgroups; **(C)** levels of some representative cytokines-(un)related to *NAMPT*/*TPI1* expression in blood. The statistical significance p values < 0.05* or 0.01** were calculated between groups matched by lines.

Above clues suggest that although *TPI1* and *NAMPT* dominant different stages of RA, they similarly promote innate immunity-mediated pathological changes. More clinical samples were used to validate this conclusion. Average age of the RA patients was 55.3 ± 12.9, and 16 of the 81 patients were male. Serological levels of RFn and hs-CRP were 267.2 ± 274.7 and 13.6 ± 26.0 pg/ml, respectively. Detailed information about these patients is included in **Supplementary S5**. The samples were equally divided into 3 groups, namely *NAMPT* ^++^, *TPI1* ^++^ and *NAMPT* ^+^ *TPI1* ^+^ subsets. Because *NAMPT* ^++^ *TPI1* ^++^ cases were not common, they were combined into *NAMPT* ^++^ subset, which made this group showed higher levels of *TPI1* than *NAMPT* ^+^ *TPI1* ^+^ controls (**Figure 8A**). The patients overexpressing *NAMPT* were with relative high levels of lymphocytes count. Of note, both *NAMPT* ^++^ and *TPI1* ^++^ subsets were diagnosed with higher levels of RFn (**Figure 8B**). In the following ELISA assay, we firstly determined CTACK, Eotaxin, and RANTES, 3 differential cytokines identified above. Although the difference about CTACK among groups was not statistically significant, higher levels of this cytokine was observed in *NAMPT* ^++^ and *TPI1* ^++^ samples. Overexpression of either *TPI1* or *NAMPT* was revealed to be related to the increased Eotaxin and decreased RANTES (**Figure 8C**). Meanwhile, we detected IL-2R, LIF and IL-17α. The results confirmed that levels of these cytokines showed no correlation to *TPI1*/*NAMPT* expression, which further validate the reliability of conclusion deduced from cytokines chip test.

### *TPI1* Promote M1 Polarization Probably by Interacting With HIF-1α

**Figure 9:**
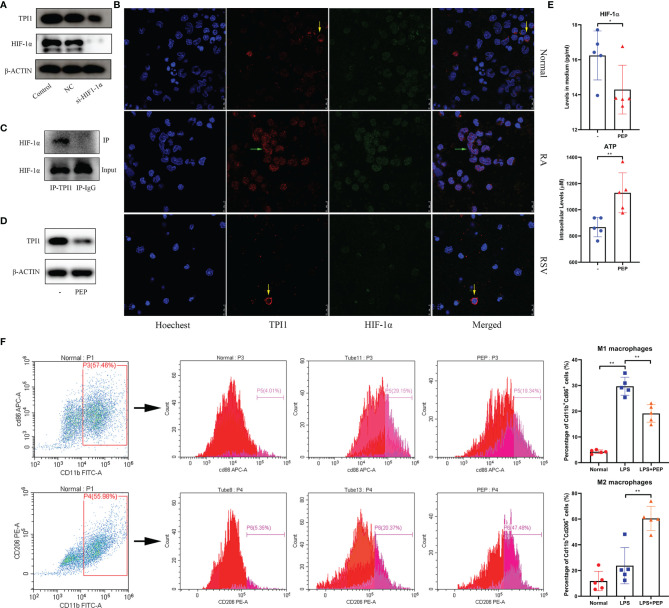
Reciprocal interactions between TPI1 and HIF-1α. **(A)** *HIF-1α* silencing impaired TPI1 expression in RA monocytes; **(B)** expression and co-localization of TPI1 and HIF-1α in normal and RA monocytes observed by immunofluorescence approach, some RA monocytes were treated by anti-hypoxia reagent RSV; **(C)** interaction between TPI1 and HIF-1α in RA monocytes revealed by Co-IP experiment; **(D)** TPI1 inhibitor PEP decreased TPI1 in RA monocytes cultured *in vitro*; **(E)** effects of PEP on intracellular ATP production and HIF-1α excretion in RA monocytes; **(F)** PEP inhibited M1 polarization of mouse peritoneal macrophages, revealed by flow cytometry analysis. The statistical significance p values < 0.05* or 0.01** were calculated between groups matched by lines.

The effects of *NAMPT* on M1 polarization have been investigated in a precious report (17). Therefore, we preliminarily investigated the role of *TPI1* in inflammation. As a glycolytic enzyme, it would be controlled by HIF-1α, the most potent inducer of aerobic glycolysis in inflammatory monocytes (23). By silencing *HIF-1α* in RA monocytes, we found that TPI1 expression was obviously down-regulated (**Figure 9A**). It implies that *TPI1* overexpression is part of inflammatory metabolism reprogramming initiated by HIF-1α. To confirm this, we performed the immunofluorescence assay. By HIF-1α accumulation, RA monocytes expressed more TPI1 than normal controls. Then, we used RSV to down-regulated HIF-1α (24). Under this situation, TPI1 became hard to be observed (**Figure 9B**). More importantly, we found that TPI1 was mainly distributed in nucleus of inflammatory cells, and it obviously co-localized with HIF-1α. When HIF-1α production was impaired, it returned to cytoplasm. This observation demonstrates possible interaction between HIF-1α and TPI1, which was evidenced by Co-IP assay (**Figure 9C**). These facts indicate that TPI1 may promote inflammation independent of its catalytic activity. As a TPI1 inhibitor, PEP decreased TPI1 in RA monocytes (**Figure 9D**). Although it inhibited HIF-1α secretion, the decrease was not very huge (**Figure 9E**). Interestingly, as a metabolic inhibitor, it did not suppress but further promote ATP production in cells. Naive mouse peritoneal macrophages were significantly activated by LPS. PEP reduced the frequency of Cd11b^+^Cd86^+^ M1 macrophages and enlarged the population of Cd11b^+^Cd206^+^ M2 macrophages (**Figure 9F**). Briefly, inhibition of TPI1 inhibited HIF-1α signaling and M1 polarization.

## Discussion

High prevalence of metabolic complication in RA patients suggests that restoring metabolic homeostasis could be similarly important as immunomodulation. The widely observed dyslipidemia encourages rheumatologist to closely monitor lipid changes in these patients and introduce medication to reduce CVD-related mortality. Interestingly, relevant treatments usually improve arthritic manifestation at the same time (25). To better understand the clinical implication of RA-related dyslipidemia, clarifying the mystery of lipid paradox is unavoidable. However, this task is very challenging. There are too many factors that should be taken into consideration, such as adipocytes and immune cells, metabolism status, secretion profile and cell functional phenotype (10). All these elements are tightly controlled by each other. Investigations on any isolated factors cannot totally address this issue. But on the other hand, regardless of the complexity, fat is simply an alternative energy resource. Altered energy requirement should serve as the foundation for any fat-related metabolic changes. From this point of view, characterizing the dynamic glycometabolism changes should be the priority to address above paradox, because glucose is the direct and most important energy resource for mammals.

Inflammation is a high energy-consuming event. Accordingly, the resting metabolic rate of RA patients is higher than healthy population, and glucose metabolism in patients suffering from inflammation is notably accelerated (26). As a result, blood glucose could be significantly reduced at inflammatory stages, just like what we observed (**Figure 1C**). This situation would then stimulate fat utilization to compensate for the increased energy expenditure and replenish blood glucose, resulting in reduced circulating lipids and intensified oxidative stress in RA patients. This theory would be helpful to explaining the decrease of triglyceride in CIA rats (**Figure 1C**). The accelerated fat oxidation has been validated by metabolomics evidence from both RA patients and rheumatic rats (27, 28). The current study further demonstrates that glycometabolism dysregulation could be a cause of the altered fat metabolism. The coordination of *SIRT1* and *PPAR-γ* determines fat turnover (29). In CIA rats, the changes of the two genes lagged behind that of *Nampt*, a glycolytic regulator (**Figures 1E**, **2C**). Meanwhile, we observed that type 2 diabetes pathway was significantly altered in CIA rats during early CIA when *Nampt* was overexpressed. Comparatively, obvious changes in fat metabolism-related pathway occurred later (**Figure 4**). Under inflammatory circumstances, glucose is mainly catabolized in the manner of glycolysis rather than aerobic oxidation because of the speed merits (30). Therefore, the status of glycolytic regulators greatly affects the severity of inflammatory diseases like RA. By controlling functions of GAPDH, NAMPT can substantially promote glycolysis, and consequently has been identified as a therapeutic target of RA (16).

In this study, we further found that TPI1 is likewise a RA-related metabolic regulator. This enzyme catalyzes the interconversion of dihydroxyacetone phosphate (DHAP) and D-glyceraldehyde-3-phosphate (G3P), and balances glycolysis and gluconeogenesis. Its deficiency greatly impairs the lifespan and function of red cells, which rely exclusively on anaerobic glycolysis for ATP supply. This situation will also increase susceptibility to infections, basically demonstrating its role in defensive immune responses (31). However, studies about its relevance with inflammation are rare. Some researchers observed that *TPI1* was overexpressed in macrophages from flamed joints, but its pathological role in RA is unclear (32). The most convincing explanation for its pro-inflammatory properties is that *TPI1* facilitates M1 polarization by promoting glycolysis. In this sense, TPI1 would coordinate with other glycolytic enzyme to finalize inflammatory metabolism reprogramming. Thereby, it is not surprising to found that its expression is controlled by HIF-1α (**Figure 9A**). However, clinical implication of its overexpression could be far away from this. Most glycolytic enzymes fuel the inflammation by providing extra ATP. Although PEP is recognized as an inhibitor of TPI1, its stimulus will not reduce ATP production, because it is a high-energy phosphate metabolite from glycolysis. Under this context, even catalytic activity of TPI1 is inhibited by PEP, overall energy metabolism is barely affected (**Figure 9E**). Our observation hints that TPI1 itself can probably control inflammatory molecular events. In inflammatory monocytes, it is translocated into nucleus. What happen in the next step is still unknown. At least, we confirm that it can bind to HIF-1α directly. Its interaction with this transcription factor gives TPI1 a chance to regulate expression of many inflammatory genes. PEP cannot slow down metabolism, but it decreased TPI1 expression (**Figure 9D**). As a consequence, the interaction between TPI1 and HIF-1α became weak, which could be the key reason for the impaired M1 polarization. To further characterize the role of TPI1 in RA, there are 2 priority issues should be addressed: 1, how does it transfer into nucleus upon inflammatory stimuli; 2, does it simply interact with HIF-1α or act as a transcription factor independently. Besides, consequences from its interaction with HIF-1α are to be thoroughly investigated. Hence, more in-depth works are needed.

Because NAMPT is believed to play a key role in RA-related inflammation, it is tricky to observe that its overexpression in CIA rats was very brief (16). Meanwhile, it occurred before the occurrence of inflammatory manifestation (**Figure 2C**). It is known that NAMPT promotes CIA progress by driving inflammatory polarization of monocytes (15, 16). But its role could be exaggerated. During most time of CIA, its overexpression was absent. Its expression was even decreased at later stages of active CIA (**Figures 1E**, **2C**). These confusing facts could be caused by the uniqueness of NAMPT. This enzyme is extensively involved in physiological functions, and controls several foundational biochemical reactions by providing NAD^+^ (14). Deficiency of NAMPT is lethal (33). Its overexpression could be similarly unsustainable, which will cause the breakdown of immune and metabolism homeostasis. Therefore, negative regulation of NAMPT would be initiated as soon as the development of inflammation. There are 2 importance clues supporting this claim. Firstly, its downstream SIRT1 exhibits typical anti-inflammatory properties. It can inhibit many inflammatory pathways through deacetylation modification (34). As a result, synthesis of inflammatory mediators is reduced, which will relieve NAMPT activation. Secondly, by inflammation progresses, intracellular ATP will be accumulated due to accelerated glycolysis. The increased ATP/ADP ratio will consequently inhibit AMPK, and ultimately abrogate NAMPT activation (35). Therefore, inflammation-related glycolysis cannot be mainly controlled by NAMPT, and *NAMPT* overexpression is only briefly observed during early stages of CIA. In this sense, NAMPT could be a passive player in inflammation. But evidences from this study also cannot solidly support above speculations. At least, it reminds us that when investigating the role of NAMPT in RA, we should not selectively focus on inflammatory stimuli. Meanwhile, many other glycolytic regulators have been confirmed to be involved in RA, like pyruvate kinase and hexokinase (36). This study further identified TPI1 as a key role involved in this process. Although they may drive inflammation by promoting glycolysis in the similar manner to NAMPT, their overexpression could be more persistent thanks to the lack of negative feedback discussed above. Consequently, simultaneous up-regulation of *Nampt* and *Tpi1* can be only observed in early CIA. It reflects the initiation of aerobic glycolysis and inflammatory responses in immune cells. Due to its sustainable overexpression, TPI1 may play an even more important role than NAMPT especially during persistent inflammation stages in RA.

Despite of some differences, evidences from this study show that both NAMPT and TPI1 affect RA manifestation by reshaping monocytes polarization. From this perspective, *NAMPT* ^++^ and *TPI1* ^++^ cases should be allocated into single one category, although they are at different disease stages of RA. Under these situations, monocytes were predominantly differentiated into M1 phenotype, resulting in accumulated inflammatory cytokines (**Figures 7**, **8**). Meanwhile, lymphocytes-released cytokines like IL-17 were largely unaffected regardless of NAMPT/TPI1 changes. Although RFn is a typical product of lymphocytes, its production can be affected by monocytes too (37). Thereby, increase of RFn in *NAMPT* ^++^ and *TPI1* ^++^ cases is reasonable. NAMPT and TPI1 seemingly mainly affect monocytes, but glycolysis is also important in deciding T cells phenotypes (38). It is irrational to exclude lymphocytes from glycolytic regulators-dominated active RA. In fact, levels of most lymphocytes-released cytokines was similar in *NAMPT* ^++^ *TPI1* ^++^ patients to *NAMPT* ^+^ *TPI1* ^+^ controls (**Supplementary S4**). Therefore, overexpression of NAMPT/TPI1 can only define a RA subtype with certain metabolic phenotype. During this period, the role of inflammatory monocyte could be predominant, but we cannot rule out influences from other immune cells. Comparatively, *NAMPT* ^+^ *Tpi1* ^+^ patients may suffer more from joints degradation due to the increased involvement of lymphocytes.

## Data Availability Statement

The original contributions presented in the study are included in the article/**Supplementary Material**. Further inquiries can be directed to the corresponding author. The RNA-sequencing data presented in the study are deposited in the GEO repository, accession number GSE192633.

## Ethics Statement

The studies involving human participants were reviewed and approved by the Institutional Ethics Research Committee of the First Affiliated Hospital of Wannan Medical College. The patients/participants provided their written informed consent to participate in this study. The animal study was reviewed and approved by the Institutional Animal Ethics Committee of Wannan Medical College.

## Author Contributions

JZ conceived this study, and wrote the manuscript. ML performed all clinical samples-related analyses. M-QT conducted the majority of animal experiments. Y-JW conducted molecular mechanism analyses. LX and X-WW collected the clinical data and samples. YL and ZY assisted all the experiments. OJO participated in the design of this study, and polished the language. All authors contributed to the article and approved the submitted version.

## Funding

This work was supported by National Natural Science Foundation of China (81973828), Major Project of Natural Science Foundation of the Department of Education of Anhui province (KJ2020A0868, KJ2019ZD32), Funding of “Peak” Training Program for Scientific Research of Yijishan Hospital, Wannan Medical College (GF2019J01).

## Conflict of Interest

The authors declare that the research was conducted in the absence of any commercial or financial relationships that could be construed as a potential conflict of interest.

## Publisher’s Note

All claims expressed in this article are solely those of the authors and do not necessarily represent those of their affiliated organizations, or those of the publisher, the editors and the reviewers. Any product that may be evaluated in this article, or claim that may be made by its manufacturer, is not guaranteed or endorsed by the publisher.
