# Supplementary material for: Metabolic Enzyme Triosephosphate Isomerase 1 and Nicotinamide Phosphoribosyltransferase, Two Independent Inflammatory Indicators in Rheumatoid Arthritis: Evidences From Collagen-Induced Arthritis and Clinical Samples
Source: Front Immunol. 2022 Jan 17;12:795626. doi: 10.3389/fimmu.2021.795626 (PMC8801790; doi:10.3389/fimmu.2021.795626)
Supplement: Supplementary file 2 [file Table_2.docx]

**Table 2** Results of cytokine chip analysis

|  | 1 | 2 | 3 | 4 | 5 | 6 | 7 | 8 | 9 | 10 | 11 | 12 | 13 | 14 | 15 | 16 | 17 | 18 | 19 | 20 |
| --- | --- | --- | --- | --- | --- | --- | --- | --- | --- | --- | --- | --- | --- | --- | --- | --- | --- | --- | --- | --- |
| mRNA NAMPT | 1.51 | 1.44 | 1.13 | 1.12 | 0.46 | 2.42 | 2.42 | 2.26 | 1.89 | 2.15 | 1.31 | 2.20 | 2.07 | 1.34 | 1.09 | 0.79 | 1.63 | 1.26 | 1.26 | 1.04 |
| mRNA TPI1 | 1.43 | 2.27 | 1.36 | 1.15 | 1.52 | 0.70 | 0.44 | 0.94 | 1.83 | 2.26 | 0.32 | 0.80 | 0.68 | 0.80 | 0.39 | 1.21 | 0.53 | 0.63 | 0.93 | 1.23 |
| CTACK.(72) | 385.5 | 451.2 | 268.89 | 339.9 | 462.20 | 907.8 | 357.2 | 273.65 | 585.6 | 846.16 | 243.4 | 287.9 | 190.59 | 451.2 | 286.32 | 358.8 | 179.2 | 213.09 | 369.8 | 289.49 |
| Eotaxin.(43) | 96.57 | 77.33 | 40.13 | 35.07 | 67.60 | 88.02 | 37.61 | 68.67 | 102.1 | 93.72 | 40.28 | 49.24 | 49.24 | 77.76 | 26.43 | 37.01 | 31.61 | 40.72 | 42.20 | 67.74 |
| Basic.FGF.(44) | 37.57 | 45.02 | 37.57 | 37.57 | 37.57 | 45.02 | 23.79 | 28.89 | 56.34 | 92.39 | 41.41 | OOR < | 60.73 | 41.41 | 147.90 | 37.57 | 28.89 | 41.41 | 37.57 | 66.26 |
| G-CSF.(57) | 66.64 | 60.70 | *29.28 | *22.44 | 84.03 | 35.87 | *15.24 | *22.44 | 141.4 | 240.75 | 42.27 | OOR < | 133.51 | 75.41 | 106.42 | *22.44 | *25.89 | 35.87 | 35.87 | 440.08 |
| GM-CSF.(34) | OOR < | OOR < | OOR < | OOR < | 7.48 | OOR < | OOR < | OOR < | *4.77 | 18.45 | OOR < | OOR < | *6.97 | OOR < | *4.17 | OOR < | OOR < | OOR < | OOR < | 39.47 |
| GRO-alpha.(61) | OOR < | OOR < | *39.83 | OOR < | OOR < | OOR < | OOR < | OOR < | 110.4 | OOR < | OOR < | OOR < | OOR < | OOR < | 366.3 | OOR < | OOR < | OOR < | OOR < | 705.3 |
| HGF.(62) | 513.3 | 646.5 | 308.2 | 296.6 | 434.4 | 546.8 | 261.4 | 445.7 | 507.6 | 766.9 | 440.1 | 284.9 | 400.3 | 513.3 | 646.5 | 377.4 | 342.9 | 383.1 | 366.0 | 1099.6 |
| IFN-alpha.2.(20) | OOR < | OOR < | OOR < | OOR < | OOR < | OOR < | OOR < | OOR < | OOR < | *12.99 | OOR < | OOR < | OOR < | OOR < | OOR < | OOR < | OOR < | OOR < | OOR < | 59.2 |
| IFN-gamma.(21) | 18.0 | 18.0 | 16.0 | 14.0 | 44.7 | 16.0 | 16.0 | 12.0 | 29.6 | 146.7 | 20.0 | 10.0 | 41.0 | 14.0 | 114.3 | 12.0 | 20.0 | 14.0 | 16.0 | 87.1 |
| IL-1.alpha.(63) | 17.6 | 17.6 | *12.37 | *12.37 | 22.8 | 15.0 | *6.95 | *6.95 | 35.5 | 77.1 | 17.6 | *1.21 | 103.2 | 28.0 | 270.4 | *12.37 | *6.95 | 22.8 | 17.6 | 147.6 |
| IL-1.beta.(39) | 3.8 | 5.0 | 2.6 | 3.8 | 9.4 | 4.6 | 3.8 | 2.1 | 7.8 | 13.6 | 5.4 | 1.3 | 3.8 | 6.1 | 8.0 | 2.1 | 2.6 | 3.2 | 3.0 | 10.5 |
| IL-1.ra.(25) | 251.6 | 270.8 | 166.5 | 189.4 | 142.1 | 211.0 | 115.7 | 101.4 | 511.8 | 877.0 | 69.5 | OOR < | 211.0 | 142.1 | 525.9 | 50.9 | 28.2 | 28.2 | OOR < | 696.1 |
| IL-2.(38) | OOR < | OOR < | OOR < | OOR < | OOR < | OOR < | OOR < | OOR < | OOR < | *3.02 | OOR < | OOR < | 21.7 | OOR < | 32.6 | OOR < | OOR < | OOR < | OOR < | 36.9 |
| IL-2.R.alpha.(13) | 45.6 | 71.3 | 62.8 | 51.4 | 48.5 | 62.8 | 35.3 | 44.1 | 68.5 | 112.3 | 82.4 | 24.6 | 65.6 | 54.3 | 44.1 | 39.7 | 48.5 | 57.1 | 39.7 | 211.3 |
| IL-3.(64) | OOR < | OOR < | OOR < | OOR < | *0.13 | OOR < | OOR < | OOR < | OOR < | 6.2 | OOR < | OOR < | 7.8 | OOR < | 23.9 | OOR < | OOR < | OOR < | OOR < | 6.7 |
| IL-4.(52) | 1.3 | 1.1 | *0.51 | *0.51 | 1.3 | 1.1 | *0.17 | *0.51 | 1.6 | 2.3 | 0.8 | OOR < | *0.51 | 0.8 | 1.1 | *0.51 | 0.8 | 1.1 | 0.8 | 7.8 |
| IL-5.(33) | OOR < | OOR < | OOR < | OOR < | OOR < | OOR < | OOR < | OOR < | OOR < | 296.7 | OOR < | OOR < | 302.3 | OOR < | *23.80 | OOR < | OOR < | OOR < | OOR < | 91.3 |
| IL-6.(19) | OOR < | 1.9 | *0.59 | OOR < | *1.41 | OOR < | OOR < | 5.4 | 8.1 | 31.3 | 7.7 | *0.92 | 20.6 | OOR < | 10.2 | OOR < | OOR < | *0.59 | OOR < | 5.4 |
| IL-7.(74) | *1.91 | 6.6 | OOR < | OOR < | OOR < | *1.91 | *1.91 | OOR < | 6.6 | 20.6 | *1.91 | OOR < | *1.91 | 6.6 | 10.5 | OOR < | *1.91 | 6.6 | OOR < | 48.5 |
| IL-8.(54) | OOR < | OOR < | *1.10 | OOR < | OOR < | OOR < | OOR < | OOR < | 16.5 | 10.6 | OOR < | OOR < | *1.10 | 5.5 | OOR < | OOR < | OOR < | *0.17 | OOR < | 27.3 |
| IL-9.(77) | 168.8 | 206.0 | 256.5 | 195.1 | 166.6 | 94.2 | 109.5 | 92.0 | 276.3 | 276.3 | 246.6 | 89.8 | 408.3 | 209.3 | 532.1 | 173.1 | 223.6 | 199.5 | 248.8 | 373.0 |
| IL-10.(56) | OOR < | OOR < | OOR < | OOR < | *1.42 | OOR < | OOR < | OOR < | OOR < | 10.8 | OOR < | OOR < | OOR < | OOR < | OOR < | OOR < | OOR < | *1.42 | OOR < | 73.2 |
| IL-12(P70).(75) | OOR < | OOR < | OOR < | OOR < | *0.93 | OOR < | OOR < | OOR < | OOR < | 63.3 | 19.8 | OOR < | 8.9 | OOR < | *3.76 | OOR < | OOR < | OOR < | OOR < | 17.0 |
| IL-12(P40).(28) | *31.54 | 81.9 | *31.54 | *0.79 | 105.3 | *0.79 | OOR < | *31.54 | 213.9 | 192.9 | 57.5 | OOR < | 254.9 | 57.5 | 631.7 | *31.54 | *0.79 | 57.5 | *0.79 | 314.9 |
| IL-13.(51) | 5.1 | 3.0 | 1.4 | 1.8 | 10.5 | *0.92 | 1.4 | 1.8 | 6.6 | 14.5 | 5.1 | OOR < | 1.4 | 7.7 | 3.3 | *0.92 | 1.4 | 3.0 | 6.8 | 27.1 |
| IL-15.(73) | OOR < | OOR < | OOR < | OOR < | OOR < | OOR < | OOR < | OOR < | OOR < | OOR < | OOR < | OOR < | OOR < | OOR < | OOR < | OOR < | OOR < | OOR < | OOR < | 527.8 |
| IL-16.(27) | 54.3 | 60.7 | 60.7 | 87.3 | 64.8 | 64.8 | 61.7 | 54.3 | 71.1 | 156.5 | 103.2 | 47.9 | 105.1 | 45.7 | 64.8 | 47.9 | 64.8 | 55.4 | 45.7 | 137.8 |
| IL-17.(76) | *5.76 | *7.16 | *4.35 | *4.35 | *7.16 | *4.35 | *4.35 | *7.16 | *7.16 | 12.6 | *7.16 | *1.43 | *7.16 | *5.76 | 14.0 | *2.91 | *4.35 | *5.76 | *5.06 | 42.5 |
| IL-18.(42) | 33.6 | 31.4 | 48.7 | 93.6 | 41.0 | 70.3 | 29.3 | 41.0 | 60.6 | 182.6 | 51.2 | 28.8 | 41.0 | 38.4 | 40.1 | 42.7 | 31.4 | 14.7 | 59.8 | 117.1 |
| IP-10.(48) | 363.2 | 471.0 | 737.6 | 445.1 | 340.4 | 443.0 | 399.1 | 346.2 | 1710 | 669.6 | 733.0 | 272.8 | 514.2 | 353.0 | 196.5 | 314.2 | 235.1 | 433.2 | 204.1 | 657.3 |
| LIF.(29) | 60.3 | 69.8 | 30.5 | 30.5 | 60.3 | 30.5 | 50.7 | 40.8 | 97.0 | 213.5 | 40.8 | *7.75 | 144.4 | 35.7 | 123.2 | 25.2 | 30.5 | 50.7 | 40.8 | 387.4 |
| MCP-1.(53) | 27.9 | 16.1 | 20.8 | 26.6 | 35.2 | 18.5 | 11.8 | 30.0 | 40.5 | 78.3 | 22.3 | 18.5 | 30.6 | 20.8 | 20.0 | 11.8 | 19.2 | 20.8 | 12.7 | 44.4 |
| MCP-3.(26) | OOR < | OOR < | OOR < | OOR < | OOR < | OOR < | OOR < | OOR < | OOR < | 6.8 | OOR < | OOR < | 4.6 | OOR < | 12.8 | OOR < | OOR < | OOR < | OOR < | 21.7 |
| M-CSF.(67) | 18.8 | 24.7 | 26.7 | 37.0 | 26.3 | 36.7 | 27.3 | 23.7 | 40.2 | 110.1 | 40.9 | 15.4 | 61.7 | 24.7 | 47.9 | 20.8 | 15.4 | 14.1 | 30.6 | 54.2 |
| MIF.(35) | OOR < | OOR < | OOR < | *27.96 | OOR < | OOR < | OOR < | OOR < | OOR < | OOR < | OOR < | OOR < | OOR < | OOR < | OOR < | OOR < | OOR < | OOR < | OOR < | *121.5 |
| MIG.(14) | 185.5 | 238.1 | 330.1 | 169.7 | 195.7 | 533.4 | 185.5 | 278.9 | 972.0 | 244.8 | 599.8 | 138.1 | 177.7 | 912.2 | 131.9 | 150.1 | 90.1 | 172.4 | 144.2 | 884.0 |
| MIP-1.alpha.(55) | 1.8 | 2.0 | 2.2 | 2.0 | 1.8 | 2.0 | 2.3 | 1.6 | 4.1 | 4.6 | 1.8 | *1.18 | 1.8 | 2.5 | 2.7 | 1.8 | 2.0 | 2.2 | 1.4 | 3.9 |
| MIP-1.beta.(18) | 104.4 | 126.8 | 158.7 | 122.1 | 93.8 | 66.7 | 81.3 | 73.8 | 155.3 | 108.8 | 143.5 | 70.3 | 188.7 | 135.8 | 223.7 | 117.5 | 146.2 | 151.5 | 155.7 | 184.0 |
| beta-NGF.(46) | OOR < | OOR < | OOR < | OOR < | OOR < | OOR < | OOR < | OOR < | 12.0 | 49.6 | OOR < | OOR < | 35.5 | OOR < | *2.44 | OOR < | OOR < | OOR < | OOR < | 33.5 |
| PDGF-BB.(47) | 209.4 | 283.9 | 338.1 | 248.5 | 156.2 | 104.4 | 93.7 | 125.4 | 296.7 | 235.5 | 202.8 | 71.7 | 482.6 | 196.2 | 209.4 | 162.9 | 369.7 | 1037.7 | 209.4 | 594.8 |
| RANTES.(37) | 624.9 | 741.7 | 1294.0 | 974.4 | 346.1 | 337.1 | 470.1 | 364.2 | 1176 | 499.8 | 1220. | 335.9 | 1890.0 | 858.8 | 1939.5 | 597.9 | 1154. | 1363.2 | 1254. | 2055.9 |
| SCF.(65) | 84.8 | 96.7 | 68.2 | 80.3 | 71.2 | 104.7 | 55.9 | 68.2 | 112.8 | 147.3 | 65.1 | 48.1 | 74.3 | 59.0 | 125.8 | 74.3 | 40.1 | 72.8 | 77.3 | 158.6 |
| SCGF-beta.(78) | 101446 | 70990 | 83386 | 80224 | 85028 | 49782 | 48734 | 67022 | 86025 | 83093. | 85966 | 59330 | 55953 | 70289 | 53741 | 69589 | 110152 | 103574 | 60728 | 98435 |
| SDF-1.alpha.(22) | 1096 | 1165 | 948.1 | 1165. | 1099.3 | 1036. | 919.4 | 1029.3 | 1092. | 1134.3 | 958.0 | 1035. | 761.5 | 1050. | 1243.8 | 1147. | 1024 | 825.3 | 992.0 | 1325.0 |
| TNF-alpha.(36) | 30.8 | 33.5 | 36.2 | 32.2 | 28.1 | 23.9 | 23.9 | 19.7 | 46.9 | 66.5 | 37.6 | 14.1 | 95.9 | 28.1 | 52.2 | 23.9 | 29.4 | 38.9 | 33.5 | 74.3 |
| TNF-beta.(30) | 144.6 | 196.9 | 243.1 | 195.3 | 160.0 | 88.2 | 99.8 | 78.9 | 253.9 | 240.0 | 229.2 | 90.5 | 383.3 | 215.4 | 520.9 | 163.0 | 210.0 | 180.0 | 229.2 | 308.0 |
| TRAIL.(66) | 63.3 | 48.3 | 57.7 | 55.9 | 36.4 | 32.2 | 34.3 | 33.3 | 77.6 | 74.1 | 55.9 | 23.6 | 78.5 | 38.4 | 74.1 | 40.4 | 52.1 | 54.0 | 42.4 | 91.4 |
| VEGF.(45) | OOR < | OOR < | OOR < | OOR < | OOR < | OOR < | OOR < | OOR < | OOR < | OOR < | OOR < | OOR < | OOR < | OOR < | OOR < | OOR < | OOR < | OOR < | OOR < | 775.0 |
