# Supplementary material for: Metabolic Enzyme Triosephosphate Isomerase 1 and Nicotinamide Phosphoribosyltransferase, Two Independent Inflammatory Indicators in Rheumatoid Arthritis: Evidences From Collagen-Induced Arthritis and Clinical Samples
Source: Front Immunol. 2022 Jan 17;12:795626. doi: 10.3389/fimmu.2021.795626 (PMC8801790; doi:10.3389/fimmu.2021.795626)
Supplement: Supplementary file 4 [file Table_4.docx]

**Sequence of primers**

| **Number** | **Primer Name** | **Sequence (5'to3')** | **Base number** | **OD** | **Purification way** | **Note** |  |
| --- | --- | --- | --- | --- | --- | --- | --- |
| 1 | *Kril*-F | CACTGTGAGGACCCTGACTT | 20 | 4 | **PAGE** | rat |  |
| 2 | *Kril*-R | GCTTTCTCTTGCCTGAGGTG | 20 | 4 | **PAGE** | rat |  |
| 3 | *Brd4*-F | AGTCCCGGGAGTACAGAGAT | 20 | 4 | **PAGE** | rat |  |
| 4 | *Brd4*-R | AACTGGCTCTTCAGGCTCAT | 20 | 4 | **PAGE** | rat |  |
| 5 | *Ddx17*-F | ACCTGCACAATGTCTCCTCA | 20 | 4 | **PAGE** | rat |  |
| 6 | *Ddx17*-R | TCAGCTCTCTAGCCTGCTTC | 20 | 4 | **PAGE** | rat |  |
| 7 | *Rac1*-F | CAGTTACACGACCAATGCGT | 20 | 4 | **PAGE** | rat |  |
| 8 | *Rac1*-R | TCTGTTTGCGGGTAGGAGAG | 20 | 4 | **PAGE** | rat |  |
| 9 | *Syk*-F | GAACACCTCCTTGGTGAGGA | 20 | 4 | **PAGE** | rat |  |
| 10 | *Syk*-R | ATAGCCCTACCCTGAGGACA | 20 | 4 | **PAGE** | rat |  |
| 11 | *Rhoq*-F | TAAGCTGATGGCCACTTGGA | 20 | 4 | **PAGE** | rat |  |
| 12 | *Rhoq*-R | TGTTCTTGCCTCACGCTCTA | 20 | 4 | **PAGE** | rat |  |
| 13 | *Gda*-F | CACCGATGTGGCTGAAGAAG | 20 | 4 | **PAGE** | rat |  |
| 14 | *Gda*-R | ATTTCCGCAAGGATCAGGGA | 20 | 4 | **PAGE** | rat |  |
| 15 | *Cxcl16*-F | CGGCTGTACAGTTTCAGAGC | 20 | 4 | **PAGE** | rat |  |
| 16 | *Cxcl16*-R | CTAGCCTCCAGACCATAGCC | 20 | 4 | **PAGE** | rat |  |
| 17 | *Cd69*-F | CTGGATTGGGCTGAGAAACG | 20 | 4 | **PAGE** | rat |  |
| 18 | *Cd69*-R | CTCCTCATAGGGAGGCCTTG | 20 | 4 | **PAGE** | rat |  |
| 19 | *Nfkbiz*-F | CATTCAGCAGCGAGTACCAC | 20 | 4 | **PAGE** | rat |  |
| 20 | *Nfkbiz*-R | GACAGTGAGTGTCGCTGAAC | 20 | 4 | **PAGE** | rat |  |
| 21 | *Tpil*-F | GTCCTGGCCTATGAACCAGT | 20 | 4 | **PAGE** | rat |  |
| 22 | *Tpil*-R | TACTTCCTGGGCCTGTTGA | 19 | 4 | **PAGE** | rat |  |
| 23 | *Cebpa*-F | GGAGCAAACATGTGCCTTGA | 20 | 4 | **PAGE** | rat |  |
| 24 | *Cebpa*-R | GGGACCTCAGCTTCCTGAAT | 20 | 4 | **PAGE** | rat |  |
| 25 | *Cebpd*-F | AACGACCGATACCTCAGACC | 20 | 4 | **PAGE** | rat |  |
| 26 | *Cebpd*-R | TAGCTTCTCTCGCAGTCCAG | 20 | 4 | **PAGE** | rat |  |
| 27 | *Loc100364062*-F | TACCTCCCAAGGGCTCCTAT | 20 | 4 | **PAGE** | rat |  |
| 28 | *Loc100364062*-R | GGCATCCTTACACAGCACAG | 20 | 4 | **PAGE** | rat |  |
| 29 | *Thbs1*-F | GATCCCAAAGGAACCTCCCA | 20 | 4 | **PAGE** | rat |  |
| 30 | *Thbs1*-R | CCAGCATAGTCGTCATCCCT | 20 | 4 | **PAGE** | rat |  |
| 31 | *Tgm2*-F | TTCCGGCTGACACTGTACTT | 20 | 4 | **PAGE** | rat |  |
| 32 | *Tgm2*-R | CAGGATCCCTCCTCCACATC | 20 | 4 | **PAGE** | rat |  |
| 33 | *Hmox1*-F | CTTACACACCAGCCACACAG | 20 | 4 | **PAGE** | rat |  |
| 34 | *Hmox1*-R | CCAGCAGCTCAGGATGAGTA | 20 | 4 | **PAGE** | rat |  |
| 35 | *Vps29*-F | AATCCCGGTGACAGGATGTT | 20 | 4 | **PAGE** | rat |  |
| 36 | *Vps29*-R | GCAAAGGTTTCCAGTGCAGA | 20 | 4 | **PAGE** | rat |  |
| 37 | *Ptprc*-F | CTGGAGGACACAGCACATTG | 20 | 4 | **PAGE** | rat |  |
| 38 | *Ptprc*-R | GCTGGTTTCTTCCGAGTCAC | 20 | 4 | **PAGE** | rat |  |
| 1 | *ACTIN*-F | TCCATCGGAGCCGAAGAAATC | 21 | 4 | **PAGE** | human |  |
| 2 | *ACTIN*-R | GTGTCGGTGGATCAAAGCACA | 21 | 4 | **PAGE** | human |  |
| 3 | *SIRT1*-F | TAGCCTTGTCAGATAAGGAAGGA | 24 | 2 | **PAGE** | human |  |
| 4 | *SIRT1*-R | ACAGCTTCACAGTCAACTTTGT | 22 | 2 | **PAGE** | human |  |
| 5 | *NAMPT*-F | CGGCAGAAGCCGAGTTCAA | 19 | 2 | **PAGE** | human |  |
| 6 | *NAMPT*-R | GCTTGTGTTGGGTGGATATTGTT | 23 | 2 | **PAGE** | human |  |
| 7 | *CD14*-F | ACGCCAGAACCTTGTGAGC | 19 | 2 | **PAGE** | human |  |
| 8 | *CD14*-R | GCATGGATCTCCACCTCTACTG | 22 | 2 | **PAGE** | human |  |
| 9 | *CD16*-F | CCTCCTGTCTAGTCGGTTTGG | 21 | 2 | **PAGE** | human |  |
| 10 | *CD16*-R | TCGAGCACCCTGTACCATTGA | 22 | 2 | **PAGE** | human |  |
| 11 | *CXCL*16-F | CCCGCCATCGGTTCAGTTC | 19 | 2 | **PAGE** | human |  |
| 12 | *CXCL*16-R | CCCCGAGTAAGCATGTCCAC | 21 | 2 | **PAGE** | human |  |
| 13 | *IL-1a*-F | TGGTAGTAGCAACCAACGGGA | 21 | 2 | **PAGE** | human |  |
| 14 | *IL-1a*-R | ACTTTGATTGAGGGCGTCATTC | 22 | 2 | **PAGE** | human |  |
|  | | | | | | | |
