# Supplementary figures and images for: Metabolic Enzyme Triosephosphate Isomerase 1 and Nicotinamide Phosphoribosyltransferase, Two Independent Inflammatory Indicators in Rheumatoid Arthritis: Evidences From Collagen-Induced Arthritis and Clinical Samples
Source: Front Immunol. 2022 Jan 17;12:795626. doi: 10.3389/fimmu.2021.795626 (PMC8801790; doi:10.3389/fimmu.2021.795626)

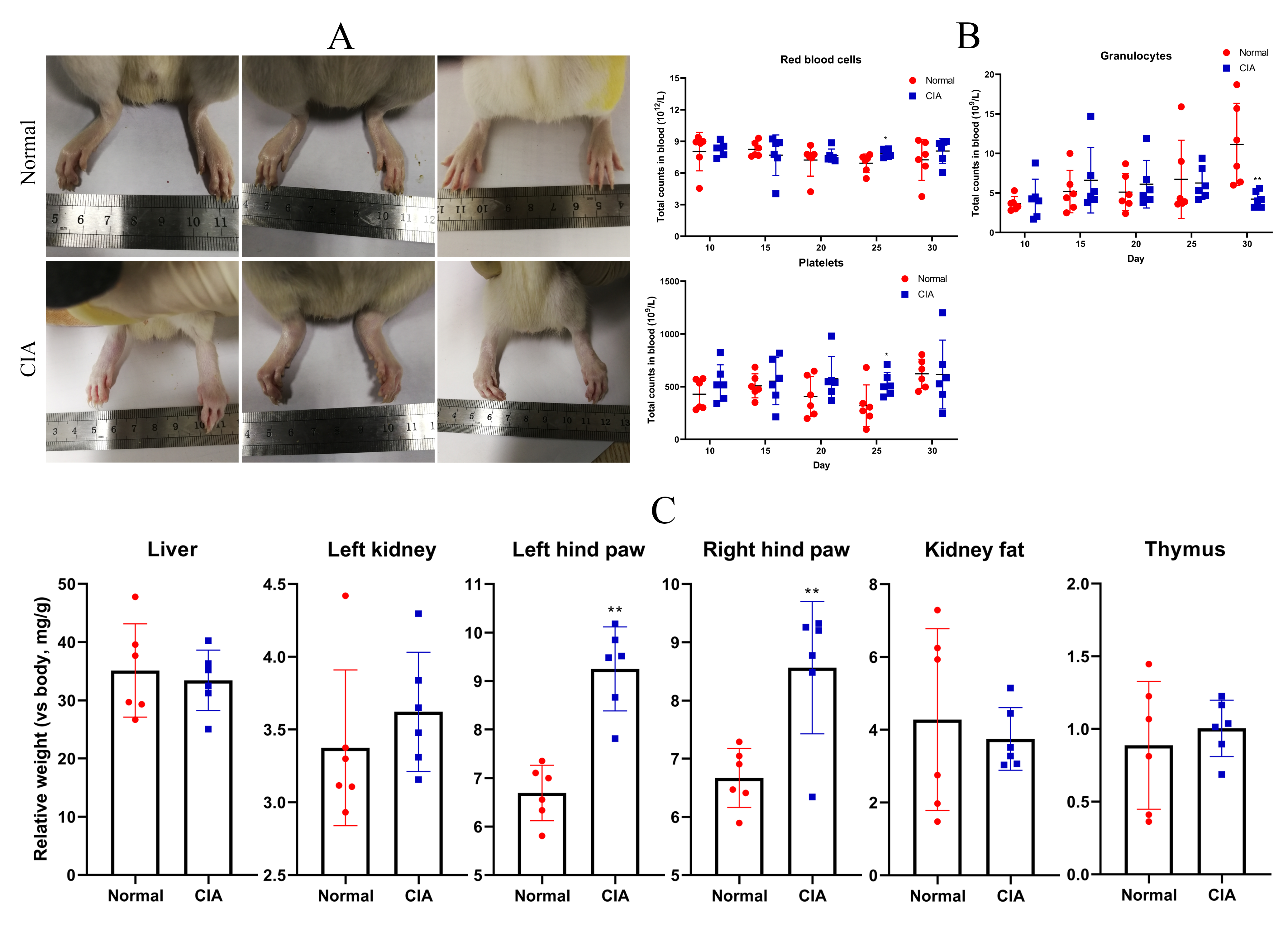

Supplement: Supplementary file 5 [file Image_1.tif]
